# Thirty percent of children and young adults with familial hypercholesterolemia treated with statins have adherence issues

**DOI:** 10.1016/j.ajpc.2021.100180

**Published:** 2021-04-02

**Authors:** Gisle Langslet, Anja K. Johansen, Martin P. Bogsrud, Ingunn Narverud, Hilde Risstad, Kjetil Retterstøl, Kirsten B. Holven

**Affiliations:** a Lipid Clinic, Oslo University Hospital, Aker Sykehus, P.O. Box 4959 Nydalen, 0424 Oslo, Norway; b Department of Nutrition, Institute of Basic Medical Sciences, University of Oslo, P.O. Box 1046 Blindern, 0317 Oslo, Norway; c Norwegian National Advisory Unit on Familial Hypercholesterolemia, Oslo University Hospital, Oslo, Norway; d Unit for Cardiac and Cardiovascular Genetics, Oslo University hospital, Oslo, Norway

**Keywords:** Familial hypercholesterolemia, Young adults, Children, Statins, Adherence

## Abstract

**Objective:**

To assess adherence to lipid lowering therapy (LLT), reasons for poor adherence, and achievement of LDL-C treatment goals in children and young adults with familial hypercholesterolemia (FH).

**Methods:**

Retrospective review of the medical records of 438 children that started follow-up at the Lipid Clinic, Oslo University hospital, between 1990 and 2010, and followed-up to the end of July 2019. Based on information on adherence to the LLT at the latest visit, patients were assigned to “good adherence” or “poor adherence” groups. Reasons for poor adherence were categorized as: “lack of motivation”, “ran out of drugs”, or “side effects”.

**Results:**

Three hundred and seventy-one patients were included. Mean (SD) age and follow-up time at the latest visit was 24.0 (7.1) and 12.9 (6.7) years; 260 patients (70%, 95% CI: 65–74%) had “good adherence” and 111 (30%, 95% CI: 25–35%) had “poor adherence”. “Lack of motivation” was the most common reason for poor adherence (*n* = 85, 23%). In patients with good adherence, compared to patients with poor adherence, age at latest visit (24.6 versus 22.0 years; *p* = 0.001), years of follow-up (13.5 versus 11.4 years; *p* = 0.003), and number of visits (8.1 versus 6.5 visits; *p*<0.001) were significantly higher, whereas LDL-C at the latest visit was lower, (3.1 (0.8) versus 5.3 (1.6) mmol/L; *p*<0.001) and percentage of patients reaching LDL-C treatment goal was higher, (34.5% versus 2.7%; *p*<0.001). Gender, BMI, age at first visit and premature cardiovascular disease in first degree relatives were not significantly associated with adherence.

**Conclusion:**

Thirty percent of young patients with FH had poor adherence to LLT, with lack of motivation as the main reason. Higher age, more visits and more years of follow-up were associated with good adherence.

## Highlights

• 30% of young patients with FH had poor adherence to statins.
• Lack of motivation was the main reason.
• Higher age, more visits and years of follow-up associated with good adherence.
• Closer follow-up and focus on patient engagement is necessary.

## Graphical abstract

**Figure:**
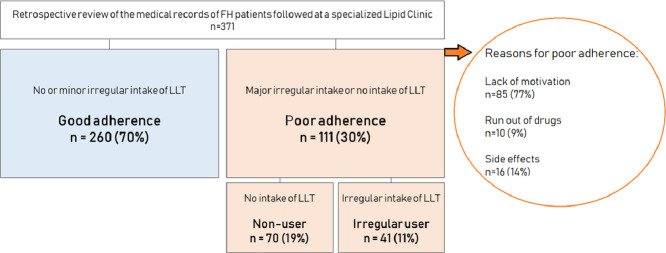

## 1 Introduction

Heterozygous familial hypercholesterolemia (FH) is an autosomal dominant condition with reduced low-density lipoprotein (LDL) receptor (LDL-R) activity, resulting in an approximate doubling of plasma LDL-cholesterol (LDL-C) levels from the first year of life. If untreated, the risk of premature atherosclerotic cardiovascular disease (ASCVD) and death is substantially increased [1]. Children with FH have increased inflammation and carotid intima-media thickness (cIMT) already from 8 years of age [2,3]. Guidelines therefore recommend lipid lowering therapy (LLT) to be initiated from around 10 years of age, with statins as the first drug of choice [4]. Early initiation of statin treatment reduces cIMT and inflammation in these children [2,5,6]. At the time of this study, the treatment goal for adults with FH without other major risk factors was LDL-C level < 2.5 mmol/L, or < 1.8 mmol/L if presence of concomitant ASCVD or other major risk factors [1]. Recently, in the updated 2019 European Society of Cardiology (ESC) and European Atherosclerosis Society (EAS) Guidelines for the management of dyslipidemias, these treatment goals have been further lowered to LDL-C level < 1.8 mmol/l and < 1.4 mmol/L, respectively [7]. For children below the age of 18 years the treatment goal is LDL-C level < 3.5 mmol/L and this has not been changed in the updated ESC/EAS guidelines [4]. Ideally, when initiated, lipid-lowering therapy (LLT) in FH patients is lifelong, except for periods of pregnancy and breastfeeding, but the treatment may be interrupted for other reasons, i.e. side effects, motivational issues or drug supply. It is well known that not only the LDL-C level, but also the duration of the LDL-C elevation (the cholesterol burden) has an impact on the atherosclerotic process [8]. Patients starting treatment later in life are at significantly higher risk than those having initiated treatment at a younger age, and adherence to the lifelong treatment is considered to be of great importance for preventing premature ASCVD [9], [10], [11]. There are some reports on treatment adherence in children and young adults with hypercholesterolemia or FH [10,12,13]. However, more knowledge is needed about the long-term adherence to LLT and the reasons for poor adherence in young individuals with FH. The aim of the present study was to assess the adherence to LLT, reasons for poor adherence and achievement of LDL-C treatment goals in a large cohort of children and young adults with FH, followed at a specialized lipid clinic. Further, we aimed to investigate associations of adherence with demographics, lipid profile, smoking habits, diet, family history, and number of visits to the lipid clinic.

## 2 Methods

**Fig. 1:**
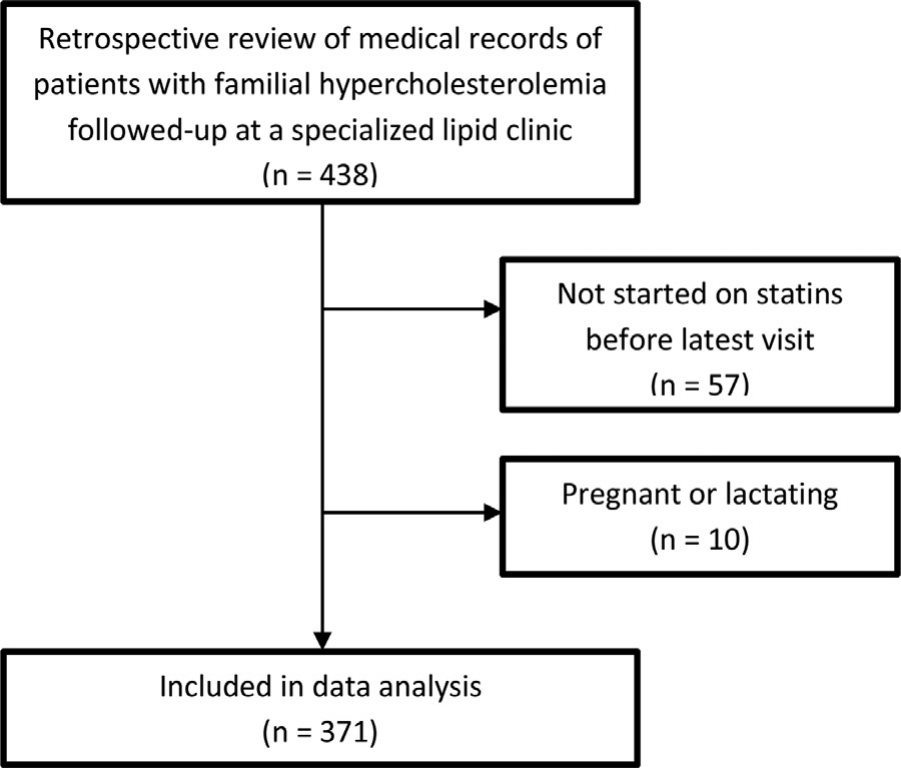
Study flow-chart.

We retrospectively reviewed the medical records of 438 children and young adults with heterozygous FH treated and followed-up at the lipid clinic, Oslo University Hospital between 1990 and 2010. Available follow-up data was collected until July 2019. Patients were included if they had at least two visits with available laboratory data. All patients were ≤18 years at the first visit to the lipid clinic, however age at first visit where cholesterol measurements were available in the medical records was above 18 years in some individuals (*n* = 9, all <23 years). We excluded those not initiated on statins, and those who were pregnant or lactating at their latest follow-up visit (Fig. 1, Study flowchart). Differences in characteristics between the excluded 67 patients and the 371 patients included in the data analysis are shown in Supplementary Table 1.

Information on adherence to LLT was collected from the latest visit, using the physicians’ assessment of how patients had used their LLT during at least the last month. Due to the fact that information was collected retrospectively there was no formal standardization of the information recorded, but review of the lipid profile and adherence to treatment are issues of special interest at every consultation. Patients were assigned to one of two groups, designated “good adherence” and “poor adherence”. The “good adherence” group was selected if there were no or minor remarks in the medical records about the regular use of LLT, and “poor adherence” was selected if there were remarks about major irregular or no use of LLT. Information about poor adherence was first collected by one of the main authors (AKJ) as quotes from the text in the medical notes. These quotes were then reviewed by the two main authors together (AKJ and GL). Reasons for poor adherence were categorized as: “lack of motivation” (including forgetfulness, carelessness and skepticism about using drugs), “ran out of drugs”, or “side effects”. All poorly-adherent patients were classified according to these three main categories. If there was evidence of more than one reason, the patient was classified according to what was considered to be the main reason. The “poor adherence” group was subdivided in two groups designated “irregular user”, if the LLT had been taken irregularly, and “non-user”, if LLT had not been taken at all.

Demographic and diagnostic data, lipid levels and other relevant blood chemistry data were also collected. The standard procedure in our clinic is that blood samples are taken at the General Practitioner and shipped to the Department of Medical Biochemistry, Oslo University Hospital, for routine analysis. From 2001, this laboratory has measured LDL-C by the direct enzymatic method. A small number of blood samples were analyzed by independent or local hospital laboratories. In most cases, the laboratory results had been analyzed in the last weeks before the visit and were available at the visit.

Diet was assessed by the validated questionnaire Smart Diet, which gives a score as a measure of the heart-healthiness of the diet, with a maximum score of 41 points [14,15]. A low score; < 27 points, indicates a non-heart-healthy diet, a middle score; 28–35 points, indicates a diet with opportunities for improvement, and a high score; ≥ 36 points, indicates a heart-healthy diet. All patients received dietary advice by a registered clinical nutritionist or a medical doctor at every visit.

In Norway, prescription medication is free of charge for children below 16 years of age, or at a fraction of the retail price when the person is above 16 years of age, through the universal public health services.

The study was approved by the Regional Committee for Medical Health Research Ethics, South East region of Norway, with permission to perform the study with passive consent. Thus, patients were given an opportunity to withdraw consent.

Continuous variables were normally distributed and are presented as means and standard deviations (SD). Categorical variables are presented as frequencies and percentages. For both continuous and dichotomous variables, a 95% confidence interval (CI) was estimated. Comparisons between groups were performed using the chi-square test or Fisher's exact test for categorical variables, depending on the expected cell frequencies. For continuous variables, comparisons between two groups and three groups were performed using the Student's *t*-test, and the one-way analysis of variance (ANOVA) with post hoc tests, respectively. When more than two groups were compared, a Bonferroni correction to the alpha level was applied to control for type 1 errors. Statistical analyses were conducted in SPSS (version 26) and STATA (version 16). All tests were two-sided. A 5% level of significance was used.

## 3 Results

Three hundred and seventy-one children and young adults were included; 57 patients not yet started on statin treatment before their latest visit and 10 patients who were pregnant or lactating were excluded from the analyses (Fig. 1, Study flowchart). Mean age (SD) at first and latest visit was 11.0 (4.0) years and 24.0 (7.1) years, respectively. Mean follow-up time was 12.9 (6.7) years, and 200 (53.9%) of the patients were male (Table 1). Mean age at initiation of LLT was 15.6 (3.5) years, reflecting that this cohort dates back to year 1990 when guidelines were different with respect to age for treatment initiation in children. All children, except one, had a confirmed pathogenic mutation in the LDL-R gene or the R3500Q mutation in the apolipoprotein *B* gene. No one had mutations in the proprotein convertase subtilisin/kexin type 9 (PCSK9) gene. One child without a confirmed mutation had elevated LDL-C (>8 mmol/L) and a first-degree relative with an FH-mutation.

**Table 1:** Demographic data.

	n	
Total number of patients, n (%)	371	
Female		171 (46.1)
Male		200 (53.9)
FH diagnosis, n (%)	371	
Clinical		1 (0.3)
Genetic		370 (99.7)
Mutation gene, n (%)	370	
LDL		364 (98.4)
APOB		6 (1.6)
Follow-up, y, mean (SD)	371	
Age first visit		11.0 (4.0)
Age latest visit		24.0 (7.1)
Years of follow-up		12.9 (6.7)

FH, familial hypercholesterolemia; y, years; SD, standard deviation; LDL, the gene encoding the LDL-receptor; APOB, the gene encoding apolipoprotein B.

Among the 57 patients that had not yet been started on LLT before their latest visit, 29 were initiated on statins at the latest visit. Among the remaining 28 patients, 12 were below 15 years of age and three attained the LDL-C goal without initiation of statin treatment. The remaining 13 patients above 15 years of age were still not started on statins despite not attaining the LDL-C treatment goal.

Almost all (370 of 371) patients on LLT were treated with statins. One patient was on monotherapy with a PCSK9-inhibitor due to statin intolerance (Table 2). In addition, 117 (32%) patients were treated with ezetimibe in combination with a statin. No patients used ezetimibe in monotherapy. Atorvastatin was prescribed in 210 (57%) patients, rosuvastatin in 126 (34%) patients, and simvastatin in 34 (9%) patients. Low or moderate-dose statin (atorvastatin 5, 10 and 20 mg, rosuvastatin 5 and 10 mg, simvastatin 10, 20 and 40 mg) was prescribed in 208 (56%) patients, and high-dose statin (atorvastatin 40 and 80 mg, rosuvastatin 20 and 40 mg, simvastatin 80 mg) in 162 (44%) patients.

**Table 2:** Prescribed lipid-lowering treatment at latest visit**.**

	n	
**Statin treatment, n (%)**	371	370 (99.7)
Any atorvastatin dose		210 (56.6)
Atorvastatin 5 mg		2 (0.5)
Atorvastatin 10 mg		58 (15.6)
Atorvastatin 20 mg		66 (17.8)
Atorvastatin 40 mg		70 (18.9)
Atorvastatin 80 mg		14 (3.8)
Any rosuvastatin dose		126 (34.0)
Rosuvastatin 5 mg		15 (4.0)
Rosuvastatin 10 mg		37 (10.0)
Rosuvastatin 20 mg		44 (11.9)
Rosuvastatin 40 mg		30 (8.1)
Any simvastatin dose		34 (9.2)
Simvastatin 10 mg		4 (1.1)
Simvastatin 20 mg		13 (3.5)
Simvastatin 40 mg		13 (3.5)
Simvastatin 80 mg		4 (1.1)
**Ezitimibe treatment, n (%)**	371	117 (31.5)
**PCSK9-inhibitor, n (%)**	371	1 (0.5)

PCSK9, proprotein convertase subtilisin/kexin type 9.

At the latest visit, 260 patients (70%, CI: 65–74%) had good adherence and 111 patients (30%, CI: 25–35%) had poor adherence to the prescribed LLT (Table 3). Among the 111 patients with poor adherence 70 were “non-users” (19% of all; 63% of the poorly-adherent) and 41 were “irregular users” (11% of all; 37% of the poorly-adherent).

**Table 3:** Adherence to statins at latest visit.

	n		95% CI
Adherence to statins, n (%)	371		
Good adherence		260 (70.1)	65.2–74.5
Poor adherence		111 (29.9)	25.5–34.8
Reasons for poor adherence, n (%)	111		
Lack of motivation		85 (76.6)	67.9–83.5
Ran out of drugs		10 (9.0)	5.0–15.8
Side effects		16 (14.4)	9.1–22.1

CI, confidence interval.

The most common reason for poor adherence was “lack of motivation” in 85 patients (23% of all 371; 77% of the 111 poorly-adherent). Side effects were noted as reason for poor adherence in 16 patients (4% of all; 14% of the poorly-adherent), and 10 patients had run out of drugs (3% of all; 9% of the poorly-adherent)

Reported side effects included myalgia, arthralgia, abdominal symptoms, fatigue/slackness, headache, exanthema, dizziness, paresthesias, thin hair and sleep disturbance. One patient had a serious side effect resulting in hospitalization due to myopathy with CK elevation to 47 000 U/L, related to intensive physical exercise, which resolved quickly. Nonetheless, all 16 patients with reported side effects were prescribed a lower dose statin or another statin, with or without ezetimibe at the latest visit. However, as per July 2019, 8 of these patients (50%) were no longer followed at the lipid clinic at own request.

Age at latest visit, years of follow-up, and number of visits at the lipid clinic were significantly higher among patients with good adherence as compared to patients with poor adherence, 24.6 and 22.0 years (*p* = 0.001), 13.5 and 11.4 years (*p* = 0.003), and 8.1 and 6.5 visits (*p*<0.001), respectively. Gender, BMI (in those ≥18 years), age at first visit and premature CVD among first degree relatives were not significantly associated with adherence. High-dose statins were used more frequently by patients with good adherence compared to those with poor adherence, 48.3% versus 33.3%, respectively (*p* = 0.009). There were fewer smokers among patients with good adherence than among patients with poor adherence, 8.3 and 15.7% respectively (*p* = 0.03), and patients with good adherence had a healthier diet, as measured by higher Smart Diet score; 32.3 and 30.8 points respectively (*p*<0.001) (Table 4).

**Table 4:** Markers related to adherence status.

	n	All	n	Good adherence	95% CI	n	Poor adherence	95% CI	P*
LDL-C, mean (SD)									
Pretreatment, mmol/L	371	6.2 (1.6)	260	6.3 (1.7)	6.1–6.5	111	6.1 (1.6)	5.8–6.4	0.20
Latest visit, mmol/L	368	3.7 (1.5)	258	3.1 (0.8)	3.0–3.2	110	5.3 (1.6)	5.0–5.6	**<0.001**
Reduction, mmol/L	368	2.5 (2.0)	258	3.2 (1.7)	3.0–3.4	110	0.8 (1.7)	0.5–1.1	**<0.001**
Reduction,%	368	36.8 (27.8)	258	48.3 (17.2)	46.2–50.4	110	9.7 (29.0)	4.3–15.1	**<0.001**
**Reaching treatment goal, n (%)**									
All	368	92 (25.0)	258	89 (34.5)	29.0–40.5	110	3 (2.7)	0.9–7.7	**<0.001**
<18 y (LDL-*C* <3.5)	56	25 (44.6)	36	23 (63.9)	47.6–77.5	20	2 (10.0)	2.8–30.1	**0.001**
≥18 y (LDL-*C* <2.5)	312	67 (21.5)	222	66 (29.7)	24.1–36.0	90	1 (1.1)	0.2–6.0	**<0.001**
**On potent statin, n (%)**	370	162 (43.8)	259	125 (48.3)	42.2–54.3	111	37 (33.3)	25.3–42.5	**0.009**
**Follow-up at lipid clinic, y, mean (SD)**									
Age first visit	371	11.0 (4.0)	260	11.2 (3.9)	10.5–11.5	111	10.6 (3.8)	9.7–11.4	0.39
Age latest visit	371	23.9 (7.1)	260	24.6 (7.5)	23.6–25.6	111	22.0 (5.4)	20.8–23.1	**0.001**
Follow-up, years	371	12.9 (6.7)	260	13.5 (7.0)	12.7–14.5	111	11.4 (5.7)	10.2–12.6	**0.003**
Number of visits	371	7.7 (4.4)	260	8.1 (4.7)	7.6–8.7	111	6.5 (3.6)	5.8–7.2	**<0.001**
**Age at statin start, mean (SD)**	371	15.4 (3.5)	260	15.6 (3.5)	15.0–15.9	111	14.8 (3.7)	14.0–15.6	0.15
**CVD risk factors**ǁ									
Premature CVD in FH parent§	232	71 (30.6)	160	51 (31.9)	25.2–39.4	72	20 (27.8)	18.8–39.0	0.64
Smoking	362	38 (10.5)	254	21 (8.3)	5.5–12.3	108	17 (15.7)	10.1–23.8	**0.03**
BMI, kg/m^2^	269	24.7 (4.7)	237	24.1 (4.5)	23.5–24.7	85	24.6 (5.2)	23.4–25.7	0.43
Smart diet score, mean (SD)	307	31.8 (3.5)	219	32.3 (3.4)	31.8–32.7	88	30.8 (3.6)	30.0–31.5	**0.001**
**Gender, n (%)**									
Male	371	200 (53.9)	260	135 (51.9)	45.9–57.9	111	65 (58.6)	49.3–67.3	0.24

BMI, body mass index; CVD, cardiovascular disease; CI, confidence interval; FH, familial hypercholesterolemia; LDL-C, LDL-cholesterol; P, p-value; SD, standard deviation; y, years.

Differences between good adherence and poor adherence were tested by 2-sample *t*-test for continuous variables and the chi-square or Fisher's exact for categorical variables, statistically significant when P < .05.

⁎ ”good-adherent” vs “poorly-adherent” patients.

ǁ n (percent) unless otherwise stated.

§ CVD <55 and <65 years of age in men and women.

In a multivariate analysis including age, number of visits, diet and smoking at latest visit as variables, diet was significantly associated with good adherence, with an odds ratio of 0.91 (CI 0.85–0.99). This indicates that for every additional score in the Smart Diet questionnaire, patients were 0.91 times less likely to be poorly-adherent to statin therapy, controlling for other factors in the model (Table 5).

**Table 5:** Association between selected variables and statin adherence.

Unadjusted		Adjusted*		
Variables	OR (95% CI)	*P*	OR (95% CI)	*P*
Age	0.95 (0.91–0.98)	**0.003**	0.96 (0.89–1.03)	0.9
Number of visits	0.90 (0.85–0.96)	**0.0062**	0.90 (0.80–1.00)	0.06
Diet	0.88 (0.82–0.95)	**0.001**	0.91 (0.85–0.99)	**0.02**
Smoking⁎⁎	2.07 (1.04–4.11)	**0.04**	2.19 (0.95–5.00)	0.06

*n* = 304.

⁎ Adjusted for the other variables included in the logistic regression model.

⁎⁎ Smoking compared to non-smoking patients.

Pretreatment LDL-C levels were similar in patients with good and poor adherence, 6.3 (1.7) and 6.1 (1.6) mmol/L, respectively (*p* = 0.200) (Table 4). In all patients, pretreatment LDL-C and LDL-C at latest visit was 6.2 (1.6) mmol/L and 3.7 (1.5) mmol/L, respectively (37% reduction from pretreatment levels). In patients with good adherence, LDL-C level at latest visit was significantly lower than in patients with poor adherence; 3.1 (0.8) and 5.3 (1.6) mmol/L (*p*<0.001), respectively (48.3% and 9.7% reduction from pretreatment levels). Within the poor adherence group, LDL-C levels were significantly lower among irregular users as compared with non-users; 4.3 and 5.9 mmol/L (*p*<0.001), respectively (22.6% and 2.0% reduction from pretreatment levels) (Supplementary Table 2).

**Fig. 2:**
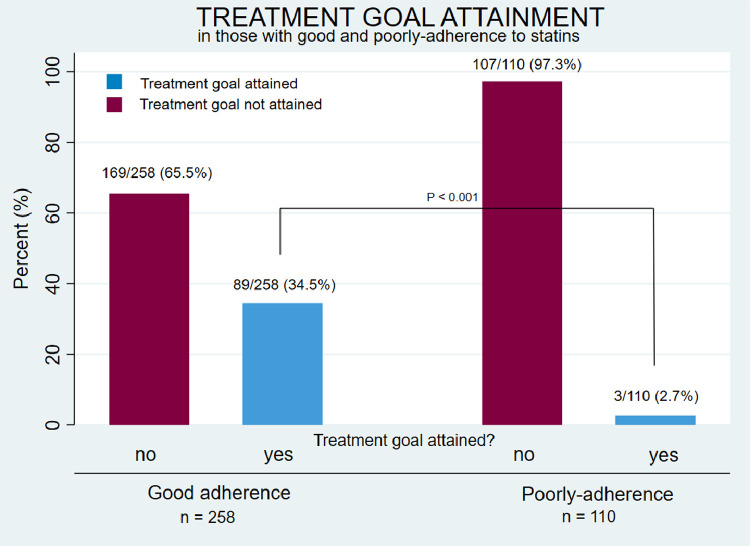
Treatment goal attainment in patients with good adherence and poor adherence.

Overall, the LDL-C treatment goals of ≤3.5 mmol/L (< 18 years of age) and ≤ 2.5 mmol/L (≥ 18 years of age) were attained by 92 (25%) patients [25 (45%) of those below 18 years of age and 67 (22%) of those 18 years and older]. Among patients with good adherence 34.5% attained the treatment goal as compared to 2.7% among patients with poor adherence (*p*<0.001) (Table 4 and Fig. 2). Among patients below 18 years of age with good adherence, 23 of 36 (63.9%) attained the treatment goal. In patients 18 years and older with good adherence, 66 of 222 (29.7%) attained the treatment goal (Table 4).

Of note, even among the 260 adherent patients at the latest visit, 82 (31.5%) patients had remarks about poor adherence in the medical records at previous visits.

## 4 Discussion

In this long-term study of children and young adults with FH followed up at a specialized lipid clinic, as many as 30% had poor adherence to their LLT. To our knowledge, the reasons for poor adherence to LLT have not been studied in detail in this group of patients previously. As expected, LDL-C levels were significantly lower, and treatment goal attainment significantly higher among patients with good adherence, confirming that the LDL-C level can be used as a marker for adherence.

Lack of motivation was the main reason given for poor adherence, apparent in 23% of all patients, and included those that had statements in their medical records of forgetfulness, carelessness/sloppiness and skepticism about using drugs, probably reflecting an underestimation of the risk associated with having high cholesterol levels over a lifespan. When including also those who had run out of drugs, a substantial number of patients (26% of all, and 86% of the poorly-adherent) had poor adherence for reasons that could have been avoided. Although the results were disappointing, it may also be seen as an opportunity to improve our communication with patients, with more focus on patient involvement and education.

Side effects were noted as the reason for poor adherence in only 4% of patients, which is lower than reported in clinical cohort studies in adults, where muscle symptoms have been reported in up to 29% of patients [16]. In children with FH, side effects have been reported in approximately 6–20% of patients [17], [18], [19].

Some of the poorly-adherent patients in our study where poor adherence were categorized as “lack of motivation” or “ran out of drugs” may therefore in fact be poorly-adherent due to side effects. Although all 16 patients with side effects were prescribed a statin or a statin plus ezetimibe at the latest visit, it is a matter of concern that eight of these patients did not show up for the next planned follow-up visit, and therefore are no longer followed at the lipid clinic at their own request. These patients have a special need for frequent consultations with advice, reassurance, and testing of alternative drug regimens.

Patients with good adherence were older at their latest visit, had more visits and more years of follow-up than patients with poor adherence, likely these factors improve adherence, but it may also be the other way around, that adherent patients adhere better to their visit appointments and treatment advice given.

Also, our finding that patients with poor adherence were more liable to smoke and had a less healthy diet than patients with good adherence, may indicate that, in simple terms, they care less about their future health, despite having knowledge of what is healthy.

Recently, Urke et al. in-depth interviewed 24 young adult individuals with FH in our lipid clinic about their thoughts on own condition and treatment, and concluded that those who “postponed the thoughts of consequences”, “belittled the treatment” and “avoided unnecessary interference” seemed to be less adherent to advice about diet and medical treatment [20].

Our results on adherence are in accordance with previously published results in children and young adults with FH, showing that up to 25% of patients were non-adherent, and in a cohort of 336 adult individuals with FH, self-reported non-adherence were present in 63% [10,12,21].

In general clinical practice, adherence to statin therapy is a common issue of concern and reports have shown poor adherence, especially in primary prevention, and when perceived risk of cardiovascular disease is low [22,23]. In several studies on different chronic conditions, more than 40% of patients on therapy for their condition have been found to be non-adherent to medical advice [24]. Adherence to life-style regimens may be even lower, with as much as 70% of patients being non-compliant [24,25].

In a recent meta-analysis of qualitative evidence, several enablers and barriers to treatment adherence in children and adults with FH were identified [26]. Important enablers identified were: Confidence in ability to successfully self-manage their condition, practical resources and support for following lifestyle treatment, and a positive relationship with healthcare professionals. Important barriers were: Concerns over the use of LLT, inadequate and/or incorrect knowledge of treatment advice, and mismatch between perceived and actual risk. Another recent investigation of the influence of patient knowledge on health-related outcomes in FH showed that insufficient knowledge of FH was negatively related to health outcomes [27]. Younger as compared to older patients with FH tend to have a lower perceived risk of CVD [28,29].

Poor adherence may be due to misunderstanding or misinterpreting, forgetting, ignoring or denying healthcare advice. In addition to clear communication, a good relationship with the patient, good knowledge and understanding of the patient`s concerns, and trust between the patient and the health care provider, are key factors in improving patient adherence [24].

Except in patients below 18 years of age with good adherence, LDL-C treatment goal attainment in our cohort was low, only 30% of patients ≥ 18 years with good adherence had an LDL-C level ≤ 2.5 mmol/L. This is in accordance with results in other cohorts of patients with FH [30], [31], [32], [33]. Considering that only 44% of our patients were on high-dose statin therapy, and only 32% were treated with ezetimibe, there are obviously potential for better utilization of these drugs, and one may also raise questions about doctors’ adherence to guidelines. However, some consultations took place several years ago, when guidelines were less strict than today, and some of the younger patients may still have been in a drug up-titration phase. To achieve the new lowered LDL-C treatment goals introduced in the 2019 EAS/ESC guidelines, many patients will need to add PCSK9-inhibitors to their statin and ezetimibe therapy.

Although somewhat disappointing, our results are likely a best-case scenario for young individuals with FH, as compared to many other countries with less universal health care systems and greater social disparities. In Norway, probably between 1/3 and 1/2 of those estimated to have FH have been diagnosed genetically [34]. Trust in the healthcare system is high, there is relative homogeneity in the population and the public health care system includes all inhabitants, with low or no costs for consultations and medicines.

Strengths of the present study are the high number of children with genetically confirmed FH and the long follow-up time in a specialized lipid clinic. Limitations are that data are based on information collected retrospectively from the medical records with no formal standardization of the information recorded. Information about adherence and time frames for adherence to LLT were approximate and based on self-reporting, which could imply even poorer adherence than reported. Also, laboratory analyses have been performed at varying time points in relation to the visits. Furthermore, we do not have information about the patients’ socioeconomic status, which may have an impact on adherence. The high number of patients and visits may, however, mitigate these weaknesses.

In conclusion, thirty percent of young FH patients followed-up for 13 years in a specialized lipid clinic had poor adherence to their lipid lowering therapy and low LDL-C treatment goal attainment. Lack of motivation was the main reason for poor adherence. Closer follow-up in children and young adults with FH is needed, with focus on patient education, support and engagement.

## Funding

This study was supported by the University of Oslo, the Norwegian National Advisory Unit on FH, Oslo University Hospital, the Throne-Holst Foundation for Nutrition Research and the South-Eastern Regional Health Authority, Oslo, Norway.

## Authors’ contributions

GL contributed to the concept and design of the study, contributed to data acquisition, and drafted the article. AKJ contributed to the concept and design of the study, acquired data, drafted the article and performed the statistical analyses. KBH and MPB contributed to the concept and design of the analysis, and made critical revision of the article for key intellectual content. IN acquired data, contributed to the concept of the analysis, and made critical revision of the article for key intellectual content. HR and KR made critical revision of the article for key intellectual content. All authors have approved the final article.

## Declaration of Competing Interest

Financial disclosure: GL reports personal fees from Amgen, Sanofi and Boehringer Ingelheim, none of which are related to the content of this manuscript. MPB reports research grants and/or personal fees from Amgen, Sanofi, MSD, Boehringer Ingelheim, Mills DA and Kaneka, none of which are related to the content of this manuscript. KBH reports grants and/or personal fees from Tine SA, Mills DA, Olympic Seafood, Amgen, Sanofi, Kaneka and Pronova, none of which are related to the content of this manuscript. KR reports personal fees from Amgen, Bayer, Chiesi, MSD, Norwegian Medical Association, The Norwegian Directorate of Health, Novartis, Sanofi, Sunovion and Takeda, and personal fees and research grants from Mills DA and from Oslo Economics, none of which are related to the content of this manuscript. AJ, IN and HR have no relevant financial relationships to disclose.

## Appendix Supplementary materials

Image, application 1
