# Supplementary material for: Thirty percent of children and young adults with familial hypercholesterolemia treated with statins have adherence issues
Source: Am J Prev Cardiol. 2021 Apr 2;6:100180. doi: 10.1016/j.ajpc.2021.100180 (PMC8315460; doi:10.1016/j.ajpc.2021.100180)
Supplement: Supplementary file 1 [file mmc1.docx]

| Supplementary Table 1. Characteristics of patients included (n=371) and excluded (n=67) in data analysis. | | | | | |
| --- | --- | --- | --- | --- | --- |
|  | **n** |  | **n** |  | ***P*** |
| **Total number of patients, n (%)** | 371 |  | 67 |  |  |
| Female |  | 171 (46.1) |  | 34 (50.7) | 0.51 |
| Male |  | 200 (53.9) |  | 33 (49.3) |  |
| **FH diagnosis, n (%)** | 371 |  | 67 |  |  |
| Clinical |  | 1 (0.3) |  | 2 (3.0) | 0.33 |
| Genetic |  | 370 (99.7) |  | 65 (97.0) |  |
| **Mutation gene, n (%)** | 370 |  | 65 |  |  |
| LDL |  | 364 (98.4) |  | 63 (96.9) | 0.54 |
| APOB |  | 6 (1.6) |  | 2 (3.1) |  |
| **Follow-up, y, mean (SD)** | 371 |  | 67 |  |  |
| Age first visit |  | 11.0 (4.0) |  | 11.3 (3.3) | 0.60 |
| Age latest visit |  | 24.0 (7.1) |  | 18.7 (6.7) | **<0.001** |
| Years of follow-up |  | 12.9 (6.7) |  | 7.4 (6.6) | **<0.001** |
| FH, familial hypercholesterolemia; y, years; SD, standard deviation; LDL, the gene encoding the LDL-receptor; APOB, the gene encoding apolipoprotein B.  Differences between included and excluded patients were tested by 2-sample t-test for continuous variables and the chi-square or Fisher's exact for categorical variables, statistically significant when P < .05. | | | | | |
|  |  |  |  |  |  |

| Supplementary Table 2 |  |  |  |  |  |  |  |  |  |  |
| --- | --- | --- | --- | --- | --- | --- | --- | --- | --- | --- |
|  |  | **Good adherence** |  | **Poor adherence** | | |  |  |  |  |
|  | **n** | **Regular user** | **n** | **Irregular user** | **n** | **Non-user** | ***P^*^*** | ***P*^‡^** | ***P*^§^** | ***P*^†^** |
| **LDL-C, mean (SD)** |  |  |  |  |  |  |  |  |  |  |
| Pretreatment, mmol/L | 260 | 6.3 (1.7) | 41 | 5.8 (1.4) | 70 | 6.2 (1.6) | 0.39 |  |  |  |
| Latest visit, mmol/L | 258 | 3.1 (0.8) | 41 | 4.3 (1.1) | 69 | 5.9 (1.6) | **<0.001** | **<0.001** | **<0.001** | **<0.001** |
| Reduction, mmol/L | 258 | 3.2 (1.7) | 41 | 1.6 (1.4) | 69 | 0.4 (1.7) | **<0.001** | **<0.001** | **<0.001** | **<0.001** |
| Reduction, % | 258 | 48.3 (17.2) | 41 | 22.6 (28.7) | 69 | 2.0 (26.4) | **<0.001** | **<0.001** | **<0.001** | **<0.001** |
| **Reaching treatment goal, n (%)** |  |  |  |  |  |  |  |  |  |  |
| All | 258 | 89 (34.5) | 41 | 2 (4.9) | 69 | 1 (1.4) | **<0.001** | **<0.001** | **<0.001** | 0.55 |
| <18 y (LDL-C ≤3.5) | 36 | 23 (63.9) | 7 | 2 (28.6) | 13 | 0 (0.0) | **<0.001** | 0.22 | **<0.001** | 0.11 |
| ≥18 y (LDL-C ≤2.5) | 222 | 66 (29.7) | 34 | 0 (0.0) | 56 | 1 (1.8) | **<0.001** | **0.001** | **<0.001** | 1.00 |
| **On potent statin, n (%)** | 259 | 125 (48.2) | 41 | 13 (31.7) | 70 | 24 (34.3) | **0.02** | 0.04 | 0.04 | 0.84 |
| **Follow-up at lipid clinic, y, mean (SD)** |  |  |  |  |  |  |  |  |  |  |
| Age first visit | 260 | 11.2 (3.9) | 41 | 10.4 (3.8) | 70 | 11.0 (4.1) | 0.46 |  |  |  |
| Age latest visit | 260 | 24.6 (7.5) | 41 | 21.7 (5.3) | 70 | 22.5 (5.7) | **0.008** | **0.003** | **0.01** | 0.47 |
| Years of follow-up | 260 | 13.5 (7.0) | 41 | 11.3 (5.7) | 70 | 11.5 (5.8) | **0.009** | 0.06 | **0.01** | 0.09 |
| Number of visits | 260 | 8.1 (4.7) | 41 | 6.3 (3.4) | 70 | 6.7 (3.7) | **0.001** | **0.003** | **0.006** | 0.56 |
| **Age at statin start, mean (SD)** | 260 | 15.6 (3.5) | 41 | 14.3 (3.6) | 70 | 15.5 (3.6) | 0.08 |  |  |  |
| **CVD risk factors^ǁ^** |  |  |  |  |  |  |  |  |  |  |
| Premature CVD in FH parent^§^ | 160 | 51 (31.8) | 28 | 6 (21.4) | 44 | 14 (31.8) | 0.53 |  |  |  |
| Smoking | 254 | 21 (8.3) | 40 | 3 (7.5) | 68 | 14 (20.6) | **0.01** | 1.00 | **0.006** | 0.07 |
| Smart diet score, mean (SD) | 219 | 32.3 (3.4) | 35 | 31.5 (2.9) | 53 | 30.2 (4.0) | **0.001** | 0.23 | **<0.001** | 0.09 |
| **Gender, n (%)** |  |  |  |  |  |  |  |  |  |  |
| Males | 260 | 135 (51.9) | 41 | 21 (51.2) | 70 | 44 (62.8) | 0.25 |  |  |  |

CVD, cardiovascular disease; CI, confidence interval; FH, familial hypercholesterolemia; LDL-C, LDL-cholesterol; P, p-value; SD, standard deviation; y, years.
Differences between regular users, irregular users and non-users were tested by ANOVA with post hoc tests for continuous variables, and the chi-
square or Fisher's exact for categorical variables. A Bonferroni correction to the alpha level was applied, setting the significance level at P < .017.
 ^*^regular user, irregular user and non-user
 ^‡^regular user vs irregular user
 ^§^regular user vs non-user
 ^†^irregular user vs non-user
 ^ǁ^n (percent) unless otherwise stated
 ^§^CVD <55 and <65 years of age in men and women
